# Endometriosis‐Associated Ovarian Carcinosarcoma Featuring Well‐Differentiated Adenocarcinoma and Fetal Rhabdomyoma‐Like Mesenchymal Components: An Unusual Case Report—With Molecular Analysis

**DOI:** 10.1155/crip/6781988

**Published:** 2026-05-27

**Authors:** Yuri Narusawa, Shiho Asaka, Tsutomu Muramoto, Saori Konno, Marina Kobayashi, Yukiko Kusama, Takeshi Uehara

**Affiliations:** ^1^ Department of Laboratory Medicine, Shinshu University Hospital, Matsumoto, Nagano, Japan, shinshu-u.ac.jp; ^2^ Department of Diagnostic Pathology, Shinshu Ueda Medical Center, Ueda, Nagano, Japan; ^3^ Department of Laboratory Medicine and Pathology, Life Science Research Center, Nagano Children′s Hospital, Azumino, Nagano, Japan; ^4^ Department of Gynecology, Nagano Municipal Hospital, Nagano, Nagano, Japan; ^5^ Department of Pathology, Nagano Municipal Hospital, Nagano, Nagano, Japan

**Keywords:** carcinosarcoma, clear cell carcinoma, ovary, rhabdomyosarcoma, seromucinous borderline tumor

## Abstract

Ovarian carcinosarcoma typically comprises high‐grade carcinoma and sarcoma components. We report a case of a woman in her 40s with endometriosis‐associated ovarian carcinosarcoma exhibiting an unusual presentation of well‐differentiated adenocarcinoma and rhabdomyosarcoma. The patient underwent bilateral salpingo‐oophorectomy and hysterectomy for tumors in both ovaries. Histologically, with a background of endometriosis, the epithelial component exhibited features of a borderline tumor with an intraepithelial carcinoma composed of Müllerian‐type epithelium in both ovaries. In the left ovary, a very minor area of clear cell carcinoma (less than 5%) was identified. In contrast, the mesenchymal component was a well‐differentiated rhabdomyosarcoma resembling fetal rhabdomyoma, constituting more than 60% of the left ovarian tumor. One year later, pulmonary metastases of the sarcomatous component were detected. Molecular analysis using next‐generation sequencing identified *PIK3CA* H1047R (c.3140A > G) and *CSF1R* (c.∗1841TG > GA) in both the epithelial and mesenchymal components, indicating a clonal origin and supporting a diagnosis of primary ovarian carcinosarcoma. The patient died 29 months after surgery despite receiving platinum‐based chemotherapy. This report highlights the challenges of diagnosing rare ovarian carcinosarcomas arising in endometriosis and with unusually low‐grade histology, and it emphasizes the need for comprehensive pathological assessment, including molecular analysis, for accurate diagnosis and optimal management.

## 1. Introduction

Ovarian carcinosarcoma, also known as a malignant mixed Müllerian tumor, is a rare and aggressive disease that accounts for 2% of ovarian malignancies. It is histologically defined as a biphasic malignancy that is typically composed of high‐grade carcinoma and sarcoma components [1]. On the other hand, endometriosis has a well‐known potential for malignant transformation, typically giving rise to epithelial carcinomas such as endometrioid carcinoma and clear cell carcinoma [2]. Endometriosis can rarely lead to the synchronous development of distinct independent tumors, such as ovarian clear cell carcinoma and uterine endometrial stromal sarcoma in the pelvic cavity [3]. However, endometriosis‐associated carcinosarcomas, in which the epithelial and mesenchymal components share a monoclonal origin, are very rare. Here, we describe an extremely rare and atypical ovarian carcinosarcoma arising in the setting of endometriosis. This tumor is characterized by well‐differentiated epithelial and mesenchymal components, notably a rhabdomyosarcoma with features resembling fetal rhabdomyoma. This case report was prepared in accordance with the Declaration of Helsinki. Ethical approval was obtained from the Nagano Municipal Hospital Ethics Committee (No. 2024‐0064). Due to the death of the patient, informed consent could not be obtained.

## 2. Case Presentation

### 2.1. Clinical Summary

**Figure 1:**
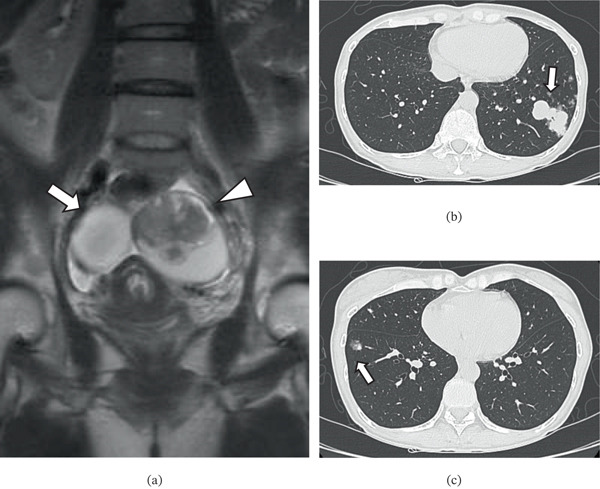
(a) Preoperative magnetic resonance imaging of both ovaries. T2‐weighted imaging shows bilateral ovarian masses with cystic and solid components (arrow: right ovary; arrowhead: left ovary). (b, c) Computed tomography images of the lungs showing multiple nodules (arrows) 1 year after the patient underwent hysterectomy and salpingo‐oophorectomy.

A woman in her late 40s with no notable medical history presented with bilateral ovarian tumors that were identified upon imaging studies. She had no notable medical or family history. Preoperative magnetic resonance imaging revealed bilateral ovarian enlargement with cystic and solid components in the left ovary (Figure 1a). She was diagnosed with a borderline ovarian tumor based on intraoperative frozen section analysis and negative cytology of intraoperative ascites. Following this, she underwent total hysterectomy, bilateral salpingo‐oophorectomy, and omentectomy. Bilateral ovarian tumors were resected intact, with no rupture of the capsules. Pelvic peritoneal endometriosis was also noted during the procedure. Histologically, the left ovarian tumor comprised two distinct components: The epithelial component exhibited features of a borderline tumor with intraepithelial carcinoma composed of Müllerian‐type epithelium; in contrast, the mesenchymal component was a well‐differentiated skeletal muscle lesion that was morphologically similar to a fetal rhabdomyoma. The right ovarian tumor was composed solely of epithelial components resembling those of the left ovarian tumor. The findings initially led to a pathological diagnosis of an ovarian seromucinous borderline tumor with intraepithelial carcinoma and a mural nodule of fetal rhabdomyoma. No adjuvant chemotherapy was administered.

One year after surgery, chest computed tomography (CT) revealed multiple nodular lesions in both lungs (Figure 1b,c). A bronchoscopic lung biopsy revealed metastasis originating from a skeletal muscle tumor; no other primary lesions were identified via imaging. Histological reevaluation of the primary ovarian tumor confirmed a final diagnosis of ovarian carcinosarcoma with a well‐differentiated heterologous rhabdomyosarcoma component. The patient subsequently received adjuvant chemotherapy with a platinum–taxane combination (six cycles of carboplatin and paclitaxel followed by three cycles of nedaplatin and paclitaxel); however, follow‐up CT revealed progressive disease. Despite further treatment modifications that included two cycles of gemcitabine monotherapy and three subsequent cycles of docetaxel monotherapy, bone metastases to the first thoracic vertebrae and malignant pleural effusion were detected. The patient died 29 months after the initial surgery.

### 2.2. Pathological Findings

**Figure 2:**
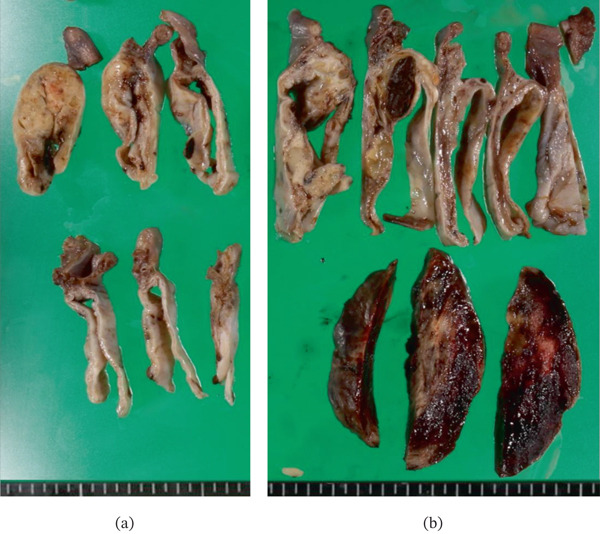
Gross pathology of both ovaries. Both (a) right and (b) left ovarian tumors exhibit cystic and solid components. The solid component of (b) the left ovarian tumor was reddish brown.

**Figure 3:**
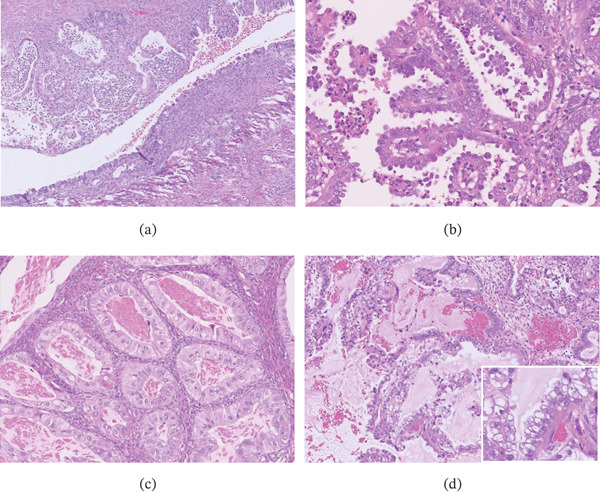
Histological features of epithelial components of the ovarian tumors. (a–d) Components common to both ovaries: (a) endometriosis, (b) papillary lesion, and (c) glandular lesion consistent with carcinoma in situ, (d) a very minor clear cell carcinoma component, constituting less than 5% of the tumor, is observed exclusively in the left ovarian tumor. The tumor cells exhibit moderate cytological atypia (inset: higher magnification).

**Figure 4:**
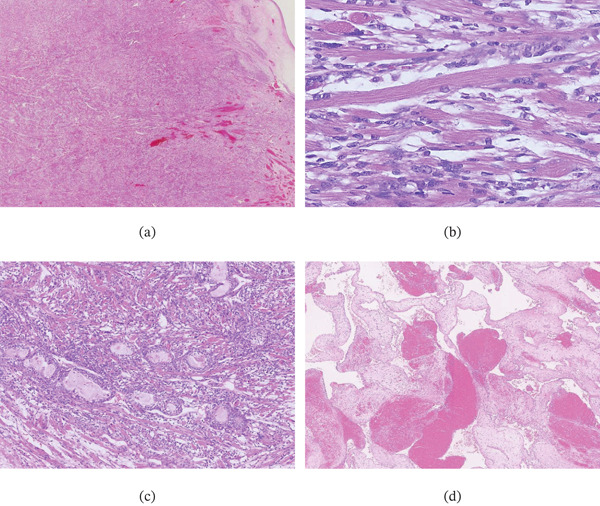
Histological features of mesenchymal components in the left ovarian tumor. (a) Fetal rhabdomyoma‐like component with well‐differentiated skeletal muscle fibers within a myxoid background, comprising more than 60% of the tumor. (b) The cytoplasm of the muscle fibers contains cross‐striations. (c) A transitional zone between the epithelial and fetal rhabdomyoma‐like components. (d) Cavernous hemangioma‐like component.

The right and left ovarian tumors measured 7 and 9 cm in diameter, respectively, and both were cystic masses with solid components (Figure 2a,b). The solid component of the left ovarian tumor exhibited intratumoral hemorrhage. Histological analysis revealed the presence of endometriosis, and both ovarian tumors were composed of various Müllerian‐type epithelial cells including columnar (with or without intracytoplasmic mucin) and ciliated cells arranged in papillary or glandular structures (Figure 3a–c). Additionally, the left ovarian mass comprised a very minor clear cell epithelial component, constituting less than 5% of the tumor. This finding is suggestive of clear cell transformation within an endometrioid tumor or a primary clear cell tumor (Figure 3d). Immunohistochemical staining revealed focal expression of both HNF‐1*β* and Napsin A in the clear cell epithelial component. Although some areas of the epithelial components showed high‐grade nuclear atypia, no destructive or infiltrative stromal invasion was observed, suggesting carcinoma in situ. Additionally, the left ovarian mass contained a solid area with a mesenchymal component consisting of fetal rhabdomyoma‐like (Figure 4a–c) and cavernous hemangioma‐like components (Figure 4d). The fetal rhabdomyoma‐like component, constituting more than 60% of the left ovarian tumor, exhibited well‐differentiated skeletal muscle fibers within a myxoid background. No necrosis, severe cytological atypia, or increased mitotic activity was observed. No teratoma components were identified in the left ovarian mass. These findings led to an initial pathological diagnosis of an ovarian seromucinous borderline tumor with intraepithelial carcinoma and a mural nodule of fetal rhabdomyoma. No metastatic lesions were identified in the uterus or omentum.

**Figure 5:**
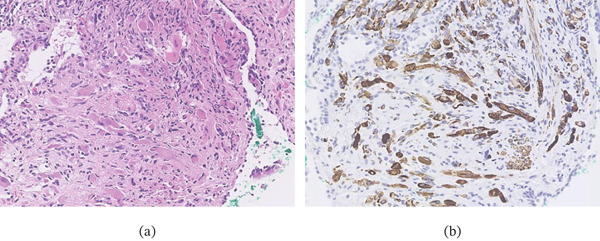
Histological images of transbronchial lung biopsies. (a) Atypical cells infiltrating the bronchial wall. (b) Immunohistochemistry demonstrating desmin positivity in the atypical cells.

Bronchoscopic lung biopsy performed a year after the initial diagnosis revealed atypical cells with abundant eosinophilic cytoplasm infiltrating the bronchial wall (Figure 5a); immunohistochemical analysis was positive for desmin (Figure 5b) but negative for alpha‐smooth muscle actin and cytokeratin AE1/AE3. The presence of rhabdomyosarcomatous differentiation combined with the absence of any other identifiable primary lesion suggested that the bronchial wall finding represented metastasis from the ovarian tumor. Histological re‐evaluation of the primary ovarian tumor confirmed the diagnosis of ovarian carcinosarcoma with a well‐differentiated heterologous rhabdomyosarcoma component.

### 2.3. Genomic Findings

To confirm the diagnosis of ovarian carcinosarcoma, targeted sequencing was performed on both the epithelial and mesenchymal components of the left ovarian mass. DNA was extracted from formalin‐fixed paraffin‐embedded tumor tissues of each epithelial and mesenchymal component using the QIAamp DNA FFPE Tissue Kit (Qiagen, Hilden, Germany) according to the manufacturer′s instructions. The library was constructed using the extracted DNA as well as the Ion AmpliSeq Cancer Hotspot Panel v2 (Thermo Fisher Scientific, Waltham, Massachusetts, United States), which targets the hotspot regions of 50 cancer‐related genes. Next‐generation sequencing was performed using an Ion GeneStudio S5 system (Thermo Fisher Scientific).

Sequencing data were analyzed using Torrent Suite Software Version 5.16.1 (Thermo Fisher Scientific). Quality control metrics of the sequencing data were strictly assessed; the mean coverage depths for the epithelial and mesenchymal components were 1709x and 1622x, respectively, and the uniformity of base coverage was 100% for both. Raw reads were aligned to the human reference genome (GRCh37/hg19) using the Torrent Mapping Alignment Program (TMAP). Initial variant calling was performed using the Torrent Variant Caller (TVC) plugin. For the precise identification of somatic mutations in the epithelial and mesenchymal components, variant filtering and annotation were performed using a customized filter chain in the Ion Reporter software. To isolate potentially pathogenic somatic mutations and filter out common germline polymorphisms, variants with a minor allele frequency (MAF) > 0.03 (3%) in population databases were excluded. The filtering criteria were designed to primarily retain protein‐altering variants, which included missense, nonsense, stop‐loss, and both frameshift and nonframeshift insertions/deletions (INDELs) or block substitutions. Synonymous variants and most noncoding variants were excluded from the final analysis, with the exception of rare noncoding variants that were shared between the components and served as critical markers for assessing clonality.

Two identical genetic alterations, a known pathogenic *PIK3CA* H1047R (c.3140A > G) mutation and a *CSF1R* variant (c.∗1841TG > GA), were identified in both the epithelial and mesenchymal components of the ovarian carcinosarcoma (Table 1). The identical mutations discovered in both components were strong evidence of a common clonal origin and supported the diagnosis of primary ovarian carcinosarcoma.

**Table 1:** Genomic findings upon analyzing the epithelial and mesenchymal components of the patient′s tumors.

Genes	Locus	Coding	Amino acid change	Variant allele frequency
***PIK3CA***	chr3:178952085	c.3140A > G	p.His1047Arg	E: 6.56%
				M: 49.00%
***CSF1R***	chr5:149433596	c.∗1841TG > GA	Not applicable	E: 13.29%
				M: 9.09%

Abbreviations: E, epithelial component; M, mesenchymal component.

## 3. Discussion

Ovarian carcinosarcoma is a biphasic malignant tumor comprising high‐grade carcinoma and sarcoma [1]. Current understanding suggests that these tumors arise from a metaplastic carcinoma, most often high‐grade serous carcinoma (HGSC), from which the sarcoma component arises [1, 4, 5]. However, the carcinosarcoma of our patient, arising in the setting of endometriosis, showed low‐grade histology of both the epithelial and mesenchymal components. Although ovarian carcinosarcoma associated with endometriosis has been reported, it is very rare [2]. This atypical presentation, with its unique origin and low‐grade features, posed a significant diagnostic challenge.

The epithelial component of the ovarian carcinosarcoma of our patient comprised Müllerian‐type epithelium that resembled the fallopian tube, endometrial, and endocervical epithelium; moreover, certain areas exhibited features of clear cell change in endometrioid borderline tumor or clear cell carcinoma. Although cytological atypia consistent with adenocarcinoma in situ was observed extensively in the Müllerian‐type epithelium of the left ovarian tumor, no obvious infiltrative/destructive invasion was observed, nor were any high‐grade adenocarcinoma components present. Although HGSC is the most common carcinoma component in ovarian carcinosarcomas, other reported types include mixed HGSC and endometrioid carcinoma; rarely, endometrioid carcinoma; and, rarely, mixed HGSC and clear cell carcinoma [1, 6]. In our patient, the absence of a high‐grade carcinoma component initially suggested a pathological diagnosis of a seromucinous borderline tumor with intraepithelial carcinoma and a mural nodule of fetal rhabdomyoma. However, the presence of a clear cell epithelial component, despite lacking the typical cytological or structural atypia of conventional clear cell carcinoma, ultimately led to the diagnosis of carcinosarcoma with adenocarcinoma (including clear cell carcinoma) and rhabdomyosarcoma components.

The sarcoma component of the patient′s ovarian carcinosarcoma was well‐differentiated, with rhabdomyosarcoma resembling fetal rhabdomyoma. Rhabdomyosarcomas are the most common heterologous components of ovarian carcinosarcomas; however, a well‐differentiated fetal rhabdomyoma‐like appearance is unusual, making it difficult to distinguish it from other benign or low‐grade lesions. In the primary surgical specimen, the rhabdomyosarcoma component showed minimal atypia, producing the differential diagnosis of a mature teratoma or mural nodule composed of rhabdomyoma as described by Huang et al. [7]. However, mature teratomas containing striated muscle components are rare, comprising fewer than 5% of cases [8, 9]. Additionally, our patient lacked other tissues of ectodermal (e.g., skin), mesodermal (e.g., adipose tissue), or endodermal (e.g., digestive tract) origin, ruling out a mature teratoma. Huang et al. [7] reported a serous cystadenoma with a mural nodule composed of a rhabdomyoma containing well‐differentiated striated muscle fibers, which resembled the histology observed in our patient. Although their patient experienced no recurrence 1 year after surgery, the limited follow‐up duration does not rule out that their patient may have harbored a carcinosarcoma with a well‐differentiated rhabdomyosarcoma component.

Molecular analysis of our patient′s tumor identified two identical genetic alterations, *PIK3CA* H1047R and *CSF1R,* in both the epithelial and sarcoma components. The *PIK3CA* H1047R mutation is a well‐known oncogenic driver documented in the Catalogue of Somatic Mutations in Cancer (COSMIC) database and has been reported in various malignancies, including breast, colon, uterine, and ovarian cancers [10]. Although the *PIK3CA* H1047R mutation is more commonly observed in ovarian epithelial tumors, it is relatively rare in ovarian carcinosarcomas. An analysis of 146 ovarian tumors registered in the COSMIC database revealed only one case of ovarian carcinosarcoma harboring this specific mutation [11]. Studies on animal models and stem cells have suggested that *PIK3CA* H1047R is associated with mesenchymal tissue differentiation [12, 13], and this mutation has also been reported in uterine carcinosarcomas [14]. Similarly, *CSF1R* mutations, which were identified in the epithelial and sarcomatous components of our patient, have been reported in ovarian carcinosarcoma [11]; as have other mutations, including those of *TP53*, *MBD3*, *PIK3CA*, *CCNE1*, *TERT*, and *MYC* [1, 15]. In the present case, although the *CSF1R* mutation (c.∗1841TG > GA) in the present case has not been reported as a pathogenic variant, it may be involved in oncogenesis. Although the direct clinical benefit of targeted therapies specifically directed at this nonpathogenic mutation may be limited, the broader implication of *CSF1R* warrants attention. The upregulation of *CSF1R* has been implicated in the pathogenesis of endometriosis [16], and recent studies have suggested the antitumor efficacy of *CSF1R* inhibitors in ovarian cancer [17]. Given the aggressive clinical course and the failure of standard chemotherapy in our patient, targeting the *CSF1R* signaling pathway could emerge as a potential therapeutic strategy for endometriosis‐associated ovarian carcinosarcomas. Finally, the absence of *TP53* mutations in our patient was consistent with the lack of an HGSC component, further exemplifying the unusual nature of the disease.

Our patient received platinum‐based chemotherapy following the diagnosis of carcinosarcoma, but she died 29 months after surgery. Although no standardized treatment strategy for ovarian carcinosarcoma exists, cytoreductive surgery followed by platinum‐based chemotherapy is the most common approach [4, 5]. Ovarian carcinosarcomas have poor prognoses, with a reported mean survival of 24 months and a 5‐year survival rate of 21% [6]. In one study, the 5‐year survival rate for ovarian carcinosarcoma was reported to be 28.2% compared with 38.4% for HGSC [18]; the difference may be attributed to the former being diagnosed often at a more advanced stage than the latter [5, 6]. The presence of a rhabdomyosarcoma component in ovarian carcinosarcomas [19] and of extraovarian metastasis of the sarcoma component [6] has been associated with poor prognosis. Although our patient initially presented with Stage 1 disease per the International Federation of Gynecological Oncology (FIGO) classification, the diagnosis of carcinosarcoma was delayed owing to the ambiguous pathological findings. Treatment was not initiated until the disease had reached Stage 4 (after the discovery of the pulmonary metastasis), which may have negatively influenced the prognosis.

Another important clinical question is whether the prognosis of endometriosis‐associated carcinosarcoma is different from that of de novo carcinosarcoma. De novo ovarian carcinosarcomas often share features with HGSC and have a very poor prognosis. On the other hand, endometriosis‐associated ovarian carcinomas have different molecular backgrounds and clinical features compared with nonendometriosis tumors [20, 21]. However, it is currently difficult to compare their prognoses directly because endometriosis‐associated ovarian carcinosarcoma is extremely rare. According to the literature, there are only five reported cases, and their follow‐up periods are generally short [22–26]. Nevertheless, it is notable that two out of the five reported cases died of the disease within a short period (6 and 10 months) [25, 26]. Our patient also showed an aggressive clinical course and died 29 months after surgery, even without high‐grade carcinoma features. We hypothesize that the high malignancy of the sarcoma component, even if it looks well‐differentiated as in our patient, overrides the relatively favorable prognosis of its endometriosis origin. More cases with long‐term follow‐up are needed to clarify this issue.

This report highlights the critical role of careful pathological assessment for the possibility of carcinosarcoma in ovarian tumors, particularly those associated with endometriosis and those exhibiting clear cell or skeletal muscle components, even in the absence of marked cytological or structural atypia. Despite the well‐differentiated histology of the ovarian carcinosarcoma of the patient, the disease progressed unexpectedly, indicating a prognosis similar to that of conventional carcinosarcomas. This suggests that prognostication based solely on histological differentiation may be unreliable in ovarian carcinosarcomas. Further investigations are required to elucidate the molecular mechanisms underlying the aggressive behavior of ovarian carcinosarcomas, particularly those with endometriosis and low‐grade histological features.

**Nomenclature**
COSMIC: Catalogue of Somatic Mutations in Cancer
CT: computed tomography
HGSC: high‐grade serous carcinoma

## Funding

No funding was received for this manuscript.

## Ethics Statement

This study was conducted in accordance with the Declaration of Helsinki and approved by the Nagano Municipal Hospital Ethics Committee (No. 2024‐0064). Due to the death of the patient, informed consent could not be obtained.

## Conflicts of Interest

The authors declare no conflicts of interest.

## Data Availability

The data that support the findings of this study are available on request from the corresponding author. The data are not publicly available due to privacy or ethical restrictions.
